# Effects of Greek Yogurt Supplementation and Exercise on Markers of Bone Turnover and Inflammation in Older Adult Exercisers: An 8-Week Pilot Intervention Trial

**DOI:** 10.3390/nu17243902

**Published:** 2025-12-13

**Authors:** Madison Bell, Pedro Henrique Narciso, Elizabeth Baker, Bareket Falk, Brian D. Roy, Andrea R. Josse, Panagiota Klentrou

**Affiliations:** 1 Department of Kinesiology, Brock University, St. Catharines, ON L2S 3A1, Canada; mb14pf@brocku.ca (M.B.); hi25lb@brocku.ca (P.H.N.); bfalk@brocku.ca (B.F.); ajosse@yorku.ca (A.R.J.); 2 Centre for Bone and Muscle, Brock University, St. Catharines, ON L2S 3A1, Canada; 3 Department of Kinesiology, McMaster University, Hamilton, ON L8S 4L8, Canada; 4 Faculty of Science, Thompson Rivers University, Kamloops, BC V2C 0C8, Canada; 5 School of Kinesiology and Health Science, York University, Toronto, ON M3J 1P3, Canada

**Keywords:** bone formation, bone resorption, interleukin 1β, interleukin 6, TNF-α

## Abstract

Background/Objectives: This 8-week randomized pilot intervention trial examined the effects of Greek yogurt (GY) supplementation on markers of bone turnover and inflammation in older adult exercisers. Methods: A total of 48 participants aged 55+ completed this 8-week intervention: 33 exercisers randomized to exercisers receiving GY (GYEX, *n* = 18, 12 females) and exercisers without GY (NYEX, *n* = 15, 12 females), and a group of 15 age-matched, community-dwelling, non-exercisers also receiving GY (GYNE, *n* = 15, 10 females). Exercisers were enrolled in a moderate-intensity community-based exercise program. GYEX and GYNE supplemented their diet with two daily servings of 175 g of GY (17 g protein, 225 mg calcium per serving). Assessments at baseline and week 8 included dietary intake, body composition, and fasting blood samples for bone markers and pro-inflammatory cytokines. Results: Body mass increased modestly across groups (time effect, *p* = 0.033), with no changes in body fat. *C*-terminal telopeptide of type I collagen (bone resorption marker) increased 14% in GYEX (time × group interaction, *p* = 0.022). Osteoprotegerin (bone formation regulator) decreased overall by 4% (time effect, *p* = 0.002). Dickkopf-1 (bone formation inhibitor) increased by 13% (*p* = 0.008) in GYNE but not in exercisers (time × group interaction, *p* = 0.018). Interleukin 1β and interleukin 6 showed significant interactions (*p* = 0.043 and *p* = 0.023), where interleukin 1β increased by 80% (*p* = 0.007) and interleukin 6 decreased by 89% (*p* < 0.001) in GYNE, but remained stable in exercisers. Tumor necrosis factor alpha remained unchanged. Conclusions: Although the observed effects of GY on the assessed biomarkers were limited and should be interpreted cautiously due to pilot design and statistical constraints, they highlight the need for longer interventions to determine whether whole-food dairy proteins can meaningfully support skeletal and immune health in older adults.

## 1. Introduction

As the global population ages, efforts to optimize bone health and mitigate inflammation are increasingly important for older adults [1]. In older populations, resistance training and aerobic exercise have positive impacts on bone health and body composition, respectively [2,3,4]. Consumption of dairy products is also beneficial for bone health in older adults [5]. In addition, according to Houston et al. (2008), consuming over 0.9 g/kg/d of protein had a greater protective effect than consuming less of this relative amount on muscle loss over a period of 3 years in older adults, with an additional protective effect observed as protein intake increased (up to 1.2 g/kg/d) [6]. Although this study did not include bone measurements, a decrease in muscle loss in this population, could, in turn, positively affect bone. However, it is currently unknown whether increased high-protein dairy food combined with multi-modal exercise can yield benefits to bone turnover in older adults.

Yogurt provides essential nutrients such as calcium and protein, which are crucial for maintaining bone health [4,7]. Evidence suggests that regular yogurt consumption is associated with improved bone mineral density (BMD) and a reduced risk of osteoporosis in older adults [8,9]. For example, Weaver et al. (2016) found that older individuals who consume dairy foods, including yogurt, exhibit higher bone mineral density (BMD) and experience significantly lower incidence of fractures [8]. Likewise, Laird et al. (2017) reported positive associations between yogurt intake and both BMD and markers of bone turnover in older adults [9]. There is also evidence that the synergistic effect of dietary calcium combined with mechanical loading through exercise can benefit bone, as shown in a group of older adults who consumed yogurt and participated in regular physical activity [10]. However, these studies have not examined whether short-term, controlled yogurt supplementation can have additive effects on markers of bone turnover in older adults already engaged in an exercise program. Because biochemical markers respond more rapidly than BMD, evaluating their early changes may provide mechanistic insights into how dairy foods influence bone remodeling before structural adaptations become apparent.

Furthermore, inflammation is a prevalent issue among older adults and plays a role in the development of chronic conditions such as osteoporosis [11]. Research has shown that yogurt, especially Greek yogurt (GY), contains bioactive peptides and bacterial cultures (sometimes probiotics) that can influence the regulation of inflammatory pathways. Specifically, one serving of GY (175 g) provides ~17 g protein (predominantly casein) and ~200–250 mg calcium, plus phosphorus and potassium [12]. Comparisons with alternative protein sources (whey, soy, pea, etc.) suggest that GY offers unique matrix advantages, including calcium bioavailability and bacterial cultures. The dairy matrix of lactose, proteins, and minerals enhances intestinal calcium absorption and deposition into bone compared with many plant sources [13]. The bacteria cultures generate bioactive peptides, organic acids, short-chain fatty acids, and exopolysaccharides, and these metabolites can strengthen tight-junction integrity, modulate immune signaling, and potentially reduce systemic inflammation [13]. Pei et al. (2017) found that daily yogurt consumption in healthy premenopausal women reduced postprandial IL-6 responses [14]. Furthermore, a 12-week randomized controlled trial in young adult males reported that adding GY to resistance/plyometric exercise training lowers pro-inflammatory IL-6 [15] while shifting bone turnover toward formation compared with an isoenergetic carbohydrate control [16]. Therefore, regular yogurt intake may promote bone health in older adults by attenuating systemic inflammation, and these effects may be further augmented when combined with regular exercise. Although there are no randomized trials specifically using GY in older adults, an 8-week intervention using 141 g of low-protein yogurt daily found no changes in several markers of bone turnover and inflammation in a group of 70 ± 9-year-old Caribbean Latino females [17]. In contrast, a review of trials examining alterative isolated whey and soy protein supplementation reported anti-inflammatory effects characterized by a reduction in circulating IL-6 and TNF-α levels, respectively, in older populations [18]. Finally, increasing habitual mixed dairy intake was associated with higher anti-inflammatory interleukin-4 and lower TNF-α, but had no effect on IL-6 in younger females with overweight and obesity [19], suggesting that anti-inflammatory effects may depend on dairy composition or population characteristics.

Taken together, the evidence suggests that it is worthwhile examining the potential bone and inflammatory responses to a high-protein dairy food such as GY in older adults, particularly those who are physically active. Therefore, the purpose of this randomized pilot intervention trial was to investigate whether GY supplementation could lead to beneficial changes in markers of bone turnover and inflammation in older adults who are regular exercisers compared to non-exercisers. We hypothesized that compared to an exercising group not consuming GY, older adult exercisers and non-exercisers supplementing their diet with GY would show positive changes in body composition and markers of bone turnover and inflammation.

## 2. Materials and Methods

### 2.1. Participants

Originally, a total of 50 male and female participants aged 55+, including 35 exercisers and 15 age-matched community-dwelling controls from the same geographical area (Niagara region, ON, Canada), were recruited to participate in this 8-week pilot intervention trial. To be included in the study, control participants were non-exercisers recruited from the community, while exercisers were required to already be actively engaged in a community-based exercise program, namely the Senior Fit program, at the Brock Functional Inclusive Training Centre for a minimum of 2 sessions per week. This criterion ensured that all exercisers had a similar activity level at baseline. All participants provided written informed consent and completed the Canadian Society for Exercise Physiology Get Active Questionnaire [20]. Participants were excluded from the study if they had an allergy to dairy foods/dairy protein or had been diagnosed with lactose intolerance. Exercising participants were also excluded from the study if they were physically injured and unable to engage with the exercise program.

The procedures of this study were approved by the Research Ethics Board at Brock University in Ontario, Canada and adhered to the ethical guidelines outlined in the latest Tri-Council Policy Statement (TCPS2) governing research involving human participants in Canada. The study was also registered on ClinicalTrials.gov (identifier: NCT06530394).

### 2.2. Experimental Design

**Figure 1:**
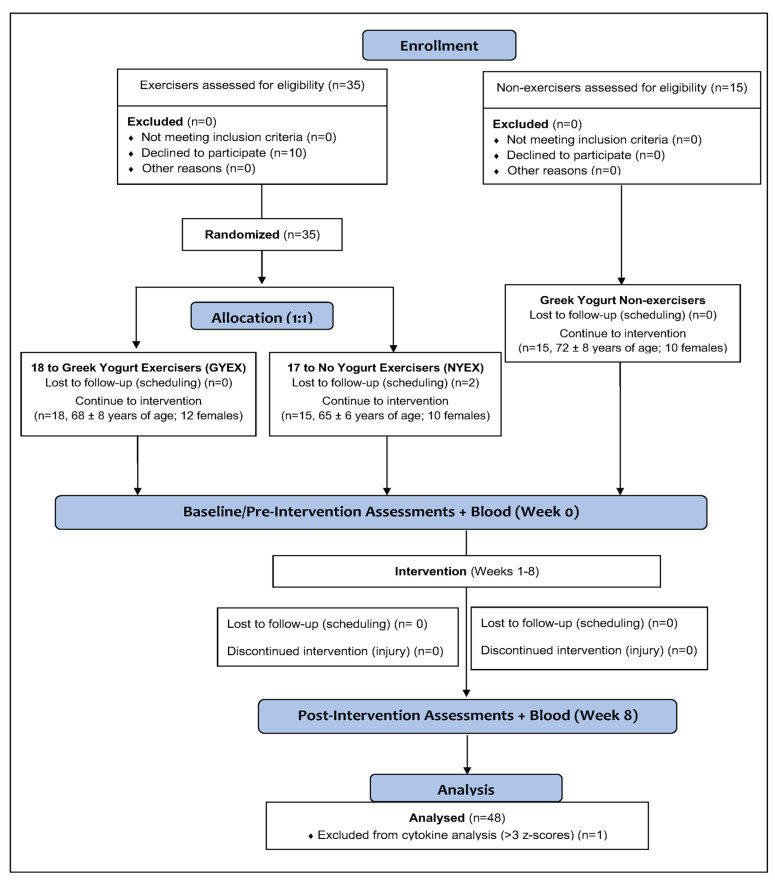
Participant flow (recruitment, randomization, completers and analysis) through the study.

This study employed an 8-week randomized controlled parallel design. To reflect real-life conditions, older male and female adults who were regular exercisers of the Senior Fit program were randomized using a computer-generated randomization sequence (1:1 allocation ratio) to one of two trial arms: either supplementing their habitual diet with GY (GYEX) or follow their typical, habitual diet (NYEX). Specifically, of the 35 exercisers, 18 were randomized to GYEX group (68 ± 8 years of age; 12 females), and 17 were randomized to the NYEX group, but 2 were lost to follow-up, thus leaving 15 participants in this group (65 ± 6 years of age; 10 females). These participants were required to continue participating in the Senior Fit program at least two times per week throughout the 8-week intervention. The Senior Fit program is a moderate-intensity, community-based program combining aerobic and resistance training. Classes are held multiple times a week and are supervised by certified exercise professionals who provide instruction, modifications, and ensure participant safety. Finally, 15 community dwelling senior adults from the Niagara community (72 ± 8 years of age; 13 females) were recruited to the non-exercisers group (GYNE) and asked to supplement their diet with GY and not enroll in any regular exercise program for the duration of the study. Figure 1 illustrates participant flow (recruitment, randomization, completers and analysis) through the study.

At the beginning (weeks 0) and the conclusion (week 8) of the study, participants completed a series of nutrition and activity questionnaires, had their body composition measured, and provided fasted, resting morning blood samples. All participants were instructed not to consume any food or liquids (except water, as needed) 8 h prior to the time of each blood draw, not to exercise for 12 h prior to the blood draw, and not to make any changes to their normal routines. These measurements took place in the Applied Physiology Laboratory at Brock University by the same investigators.

### 2.3. Intervention

NYEX participants were instructed to follow their typical, habitual diet during the 8-week period. To reflect real-life conditions, participants in the GYEX and GYNE were asked to supplement habitual diets with GY without replacing any dietary components. Specifically, GYEX and GYNE participants supplemented their typical diets with 2 servings/day (preferably morning and night) of 175 g of commercially available GY (0% MF, flavored, 130 calories, 17 g protein, 225 g calcium) for the 8-week period. The Greek yogurt provided (OIKOS High Protein GY) was a high-protein product made from skim milk, milk protein concentrate, cream, and active bacterial cultures (S. thermophilus and L. bulgaricus). For their convenience, the participants were provided with appropriate scoops to measure out a 175 g serving of GY from larger 650 g commercially available, pre-packaged containers. Although it is recommended to consume the two servings morning and night, to increase ecological validity and strengthen the feasibility of the intervention, the timing of the servings was flexible to facilitate daily routines and lifestyles. For the same reasons, participants were allowed to choose the GY flavor and were permitted to consume the GY with other foods (e.g., fruit, granola, honey). No other dietary changes were made, and no dietary counseling was provided.

The study was conducted as a single-blind trial, where researchers were not aware of group assignments. Specifically, the GY was distributed to each participant weekly by independent research assistants. Due to the short duration of the intervention (8 weeks), adherence to the supplementation was not objectively tracked but was verified verbally with each GY distribution.

### 2.4. Measurements

Anthropometric and body composition measurements were performed at the beginning (week 0) and the conclusion of the study (week 8). Body mass (kg) and body composition, including lean body mass (LBM), fat mass (FM), and body fat percent (%BF), were measured using bioelectrical impedance analysis (BIA; InBody520, Biospace Co., Inc., Los Angeles, CA, USA) following standard procedures. Briefly, participants were instructed to consume 1 cup of water before their lab visit and to void their bladder before the BIA assessment. Height was measured using a stadiometer (Seca 213 Portable Stadiometer, CME Corp., Warwick, RI, USA).

Participants’ energy intake and expenditure, including dietary intake, as well as macronutrient, micronutrient, vitamin, and supplement intakes were recorded once at each of weeks 0 and 8 using the Automated Self-Administered 24-h Dietary Recall (ASA24) [21]. Participants completed the ASA24 recalls with guidance from the researchers to ensure accuracy and consistency.

At the same assessment points (weeks 0 and 8), 10 mL of blood was collected from an antecubital vein by a certified phlebotomist or registered nurse using a standard venipuncture technique. All blood samples were left at room temperature for 10 min before being centrifuged at 1405× *g* at 4 °C for 10 min. Serum and plasma were aliquoted into pre-labeled Eppendorf tubes and stored at −80 °C until analysis. Serum was then analyzed for markers and regulators of bone turnover and pro-inflammatory cytokines by Eve Technologies Corp. (Calgary, AB, Canada). The analyses of amino-terminal propeptide of type I collagen (P1NP) and *C*-telopeptides of type I collagen (CTX-I) were performed on a PerkinElmer EnSpire™ 2300 Multilabel Reader System (Waltham, MA, USA). P1NP was measured using the Human P1NP ELISA Kit (Elabscience Biotechnology Inc., Houston, TX, USA) with assay sensitivity of 9.38 pg/mL and an intra- and inter-assay coefficient of variation (%CV) < 10 and <15, respectively. CTX-I was measured using the Human CTX ELISA Kit (Elabscience Biotechnology Inc., Houston, TX, USA) with assay sensitivity of 0.09 ng/mL and an intra- and inter-assay %CV < 10 and <15, respectively. Osteocalcin (OC), osteoprotegerin (OPG), sclerostin (SOST), dickkopf-1 (DKK-1), parathyroid hormone (PTH), interleukin 1β (IL-1β), interleukin 6 (IL-6) and tumor necrosis factor alpha (TNFα) were simultaneously measured using a Human Bone Magnetic Bead Multiplex Assay (MilliporeSigma, Burlington, MA, USA) on the Luminex™ 200 system (Luminex Corporation, Austin, TX, USA) with Bio-Plex Manager™ software version 6.1 (Bio-Rad Laboratories Inc., Hercules, CA, USA). Assay sensitivities of these markers range from 1.9 to 68.5 pg/mL, the intra-assay %CV was <10 and the inter-assay %CV was <15. Receptor activator of nuclear factor kappa-Β ligand (RANKL) analysis was performed using the MESO^®^ QuickPlex SQ 120MM Instrument with Methodical Mind and DISCOVERY WORKBENCH^®^ Software version 4.0 (Meso Scale Discovery, Rockville, MD, USA). Concentrations were measured using the Human MSD^®^ RANKL custom assay (Rockville, MD, USA) with assay sensitivity of 1.8 pg/mL. Samples passed internal quality checks, and duplicates met consistency thresholds with intra- and inter-assay %CV < 10 and <15, respectively.

### 2.5. Statistical Analysis

Statistical analyses were conducted using SPSS (Version 28.0; IBM Corp., Chicago, IL, USA). Two-way repeated measures analysis of variance (RMANOVA) was used to assess the main effects of time (within-subjects), and group (between-subjects; three levels: GYEX, GYNE, NYEX), and their time × group interactions for dietary intake and body composition. Due to the high intra-individual variability at baseline, we used linear mixed models to evaluate the main effects of time (repeated measures), and group, along with their interactions, on markers of bone turnover and inflammatory cytokines. These models included baseline concentrations of the analyte as covariate (fixed effect), and participant ID as a random effect. For significant effects, post hoc analyses were carried out, and *p*-values were adjusted for multiple comparisons using false discovery rate (FDR) correction. Statistical significance was set at *p* ≤ 0.05.

## 3. Results

### 3.1. Dietary Intake and Body Composition

As shown in Table 1, analysis of dietary intake revealed clear effects of GY supplementation on protein and calcium intake during the 8 weeks of the intervention. Protein intake significantly increased over time in GYEX and GYNE, while in the NYEX group slightly declined (Table 1). A similar pattern was observed for daily energy, calcium and vitamin D intake, which also increased significantly over time in both GYNE and GYEX, whereas NYEX participants experienced a reduction (Table 1).

There was a significant main effect for time for body mass, which significantly increased over time across groups (F = 4.87, *p* = 0.033), with a small but statistically significant overall gain of 0.49 kg from week 0 to week 8 (Table 2). While this increase was only evident in the GY groups, statistically the time × group interaction was not significant, and there was no main group effect. In contrast, %BF did not significantly change over time, nor was there a time × group interaction. However, a significant main group effect was observed (F = 5.69, *p* = 0.006). Post hoc comparisons revealed that participants in the GYNE group had consistently higher %BF compared to both the GYEX (*p* = 0.010) and NYEX (*p* = 0.026) groups, with mean differences of approximately 9% and 8%, respectively. No significant difference in %BF was found between GYEX and NYEX (Table 2).

### 3.2. Markers and Regulators of Bone Turnover

There were no significant main time effect, group effect or time × group interaction observed in OC or P1NP, markers of overall turnover and bone formation, respectively (Table 3). The bone resorption marker CTX-I demonstrated a significant time × group interaction (F = 4.00, *p* = 0.022, η_p_^2^ = 0.09), reflecting a significant 14% (*p* = 0.007) increase in CTX-I from week 0 to week 8 only in the GYEX group (Table 3).

OPG, an osteokine that inhibits bone resorption, showed a significant main time effect (F = 10.58, *p* = 0.002, η_p_^2^ = 0.11), with no main group effect and no time × group interaction (Table 3), reflecting an overall 4% decrease from week 0 to week 8 in all groups collapsed. RANKL, an up-regulator of bone resorption, and SOST, a down-regulator of bone formation, showed no significant time effect, group effect or time × group interaction (Table 3). DKK-1, also an osteokine inhibiting bone formation, showed a significant main time effect (*F* = 5.81, *p* = 0.018, η_p_^2^ = 0.07) and a significant time × group interaction (*F* = 4.01, *p* = 0.021, η_p_^2^ = 0.08) with no main group effect (Table 3). The interaction reflects a group specific response characterized by a significant +13% (*p* = 0.008) increase from week 0 to week 8 in GYNE, an +8% increase in NYEX (*p* = 0.092) but a −3% decrease in GYEX (*p* = 0.490). Finally, PTH showed no significant main time or group effects, or time × group interaction (Table 3).

### 3.3. Inflammatory Cytokines

For IL-1β, there was a significant time × group interaction (F = 3.41, *p* = 0.043, η_p_^2^ = 0.14) with no main effects of time or group (Table 4), reflecting a significant increase in IL-1β concentrations from week 0 to week 8 in the GYNE group (+89%, *p* = 0.007), a non-significant increase in the NYEX (+43%, *p* = 0.938) and a non-significant decrease in the GYEX (−39%, *p* = 0.679). The opposite pattern was observed in IL-6, where there was also a significant time × group interaction (F = 3.97, *p* = 0.023, η_p_^2^ = 0.11) with a significant main time effect (F = 12.39, *p* < 0.001, η_p_^2^ = 0.16), but no main group effect (Table 4), reflecting an overall decrease in IL-6 from week 0 to week 8, which was significant in the GYNE group (−86%, *p* < 0.001), but did not reach significance in the NYEX (−46%, *p* = 0.156) and GYEX (−76%, *p* = 0.649) groups. In contrast, TNF-α levels remained stable, with no significant main effect of time, or group, and no time × group interaction (Table 4).

## 4. Discussion

This 8-week pilot intervention trial investigated the effects of GY supplementation on markers of bone turnover and inflammation, along with changes in body composition, in older adult exercisers compared to non-exercisers. The GY supplementation successfully increased bone-supportive nutrient intake but produced limited physiological effects within this short timeframe. As this was a relatively short (8 weeks) pilot intervention in older adults who were already exercising, and as we did not control for the intensity of the exercise program, the observed effects were potentially confounded by exercise or time. Thus, the findings can only be considered preliminary. Notably, the absence of significant time × group interactions for several biomarkers—likely due to type II error—indicates that causality between GY and biomarker changes remains unproven. Future research studies should consider a longer dairy supplementation, and a tightly supervised/controlled exercise program.

### 4.1. Dietary Intake and Body Composition

As intended, GY supplementation increased protein and calcium intake in the GYNE and GYEX groups, while intake remained lower in the NYEX group. These changes confirm dietary adherence to the nutritional intervention and facilitate greater nutrient exposure that could, in theory, support musculoskeletal health in older adults. Houston et al. (2008) demonstrated that older adults consuming over 0.9 g/kg/d of protein have a greater protective effect on muscle loss than those consuming less than 0.9 g/kg/d over a period of 3 years, with an additional protective effect as protein intake increases (up to 1.2 g/kg/d) [6]. Although our participants in the yogurt groups increased their protein intake to 1.1–1.3 g/kg/d, this increase did not induce any significant measurable change in body composition. Indeed, body mass and body composition remained largely unchanged in both exercisers and non-exercisers irrespective of whether they supplemented their diet with GY or not. The small but statistically significant increase in body mass observed across all groups occurred uniformly and likely reflects normal, short-term fluctuations in hydration, or day-to-day dietary variability rather than a physiologically meaningful change in fat or lean tissue. These findings suggest that short-term increases in dairy protein do not meaningfully alter body composition in older adults beyond what is attributed to regular exercise.

Our results are consistent with those of Sandby et al. (2024), who conducted a 3-week randomized crossover trial in adults and older adults comparing two fermented (yogurt and sour milk) and two non-fermented (milk and cream) dairy products, each providing approximately 45 g of dairy protein per day [22]. Although their intervention was shorter and did not include exercisers or an exercise component, there were no changes in body composition across treatments, although all intervention conditions positively affected metabolic risk markers including blood pressure, fasting insulin and *C*-peptide, HOMA-IR, blood lipids, and alanine aminotransferase [22]. Similarly, a 12-week intervention of protein-enriched food consumption and exercise did not lead to improvements in body composition but did improve measures of muscle function and glucose metabolism marker profile (i.e., insulin sensitivity, lipid profile, and HOMA-IR) in middle-aged and older adults [23,24]. However, these effects were mostly attributed to the exercise component rather than the protein supplementation [25]. Interestingly, dairy and protein interventions combined with exercise in younger populations have shown a positive impact in reducing body fat and/or increasing lean mass [12,26,27,28], suggesting that the responses to whole-food dietary interventions may be age-specific, or due to higher exercise intensity applied to the younger populations, longer intervention (12 weeks versus 8 weeks in our study), and/or to the higher dairy intake in these studies.

### 4.2. Markers and Regulators of Bone Turnover

CTX-I, the primary marker of bone resorption, only increased in the exercisers consuming GY (i.e., GYEX group). Although small, this change was contrary to expectations, as studies of longer duration have reported a reduction in resting CTX-I concentrations following either 1 year of exercise intervention [29] or 7 months of dairy supplementation alone [30]. Although CTX increased in the GYEX group, no corresponding rise in P1NP or OC was observed, a pattern consistent with the sequence of bone remodeling, where resorption precedes formation. Bone formation cannot begin until sufficient osteoprogenitors accumulate at the resorption site; however, this recruitment and expansion process slows with aging, often spanning several months [31]. Our 8-week intervention in older adults may have been too short to detect resting increases in bone formation markers, with the rise in CTX-I among exercisers instead likely reflecting the early resorptive phase of newly initiated remodeling cycles before detectable formation responses could emerge. Josse et al. (2012) reported an increase in P1NP, but not CTX-I, following a 4-month dairy intervention paired with frequent aerobic and resistance exercise in younger women (19–45 yr), a pattern that differs from our findings and likely reflects the longer duration of the trial (16 weeks vs. 8 weeks) as well as age-related differences in remodeling dynamics, since older adults typically exhibit slower formation responses and a prolonged resorptive phase when initiating new remodeling cycles [32].

Furthermore, OPG, a decoy receptor that inhibits RANKL-mediated osteoclastogenesis, decreased over the 8 weeks across groups, likely reflecting an aging effect, while RANKL levels remained unchanged. This is a surprising finding as a previous study in postmenopausal women, found OPG significantly increased after a 30-month dairy protein intervention [30]. Moreover, increased dairy and protein consumption has been shown to decrease RANKL concentrations in exercising premenopausal women following a 4-month intervention [32]. Collectively, these findings suggest that the short-duration dairy intervention in the present study was insufficient to elicit meaningful changes in bone remodeling regulators of the OPG/RANKL pathway in older adults.

Conversely, DKK-1, a Wnt pathway inhibitor that downregulates bone formation, showed a significant time × group interaction, reflecting a significant increase from week 0 to week 8 in the non-exercisers, a lesser, non-significant increase in the NYEX while it remained unchanged in the GYEX group. This suggests a potential synergistic effect of GY and exercise in suppressing the catabolic DKK-1 in the exercising older adults compared to non-exercisers. The short-term effect of dairy proteins on the Wnt signaling inhibitors of bone formation—including DKK-1 and SOST—is less explored; therefore, more studies are needed to confirm the effects on DKK-1 [33]. Finally, contrary to our expectations, no significant changes in PTH were observed across groups. PTH is mainly responsible for regulating calcium homeostasis in blood and is positively associated with bone resorption. Previous dairy interventions have consistently reported reductions in circulating PTH as a result of increased calcium intake in younger adult populations [26,30,32,34]. In contrast, our pilot GY intervention produced only modest effects on markers and regulators of bone turnover in both older exercisers and non-exercisers, suggesting potential age-specific mechanisms. However, longer and more comprehensive interventions are required for meaningful changes in both resorption and formation to occur in older adults, thereby confirming these observations and clarifying the mechanistic basis of potential bone benefits from dairy consumption in this population.

### 4.3. Inflammatory Cytokines

Inflammatory outcomes showed an increase in IL-1β in the non-exercisers, while it remained relatively unchanged in the exercisers, particularly in the GYEX. Previous trials examining IL-1β have shown no effect of dairy consumption alone in 20–45-year-old males and females with overweight and obesity [35] or in combination with exercise in 18–20-year-old males [15]. In the study by Fraschetti et al. (2025), although a significant reduction in IL-1β was observed after 12 weeks of resistance exercise training in their young males, this effect was likely driven by the consistent decrease in the carbohydrate supplementation group [15]. In contrast, IL-1β levels in their GY supplementation group declined after one week but returned to baseline after 12 weeks [15]. Thus, our finding of an increase in IL-1β levels following 8 weeks of GY supplementation in the older non-exercisers is puzzling and warrants further investigation. Nevertheless, the observation that IL-1β remained unchanged in GYEX, unlike the increase noted in the other groups (significantly only in GYNE), may point to a potential synergistic anti-inflammatory effect of GY when combined with exercise in older adults.

IL-6 was higher at week 0 in the non-exercisers compared to our exercisers, likely due to their higher %BF, at the beginning of the intervention [36,37]. However, IL-6 significantly decreased post-intervention in this group, suggesting that GY exerted an anti-inflammatory effect, particularly in those who needed it most. Importantly, the 89% reduction in IL-6 observed in GYNE appears clinically meaningful, as baseline IL-6 concentrations in this group were elevated relative to values previously reported in healthy, normal-weight older adults of <2 pg/mL [1,8]. Given that elevated resting IL-6 is linked to greater systemic chronic inflammation, adiposity, and chronic disease risk [36,37], lowering its levels toward ranges associated with lower systemic inflammation risk suggests a potentially beneficial shift in inflammatory status, independent of exercise. However, previous work by Van Meijl and Mensink (2010) reported that low-fat dairy consumption did not affect IL-6 concentrations in overweight individuals [38]. Likewise, Gjevestad et al. (2017) reported no changes in IL-6 after 12 weeks of protein-enriched milk supplementation in older adults [39]. In contrast, Fraschetti et al. (2025) observed a reduction in IL-6 following GY consumption combined with resistance exercise in young males, suggesting that exercise may be necessary to enhance the anti-inflammatory effects of dairy proteins [15]. This suggestion aligns with the non-significant drop in IL-6 within our exercisers and supports the idea that anti-inflammatory effects may depend on acute timing (e.g., pre-load ingestion) or on specific population characteristics rather than long-term dairy intake alone. Thus, the lack of change in the GYEX group likely reflects a stabilization of an anti-inflammatory influence of habitual exercise rather than an absence of response to GY supplementation.

TNF-α remained unchanged from week 0 to week 8 across all our groups. Consistent with our findings, Fraschetti et al. (2025) reported no change in TNF-α following a GY intervention combined with resistance training in young males [15]. Another study in older adults examined the inflammatory effects of 12 weeks of protein-enriched milk compared to an isocaloric drink and, in line with our results, found no change in TNF-α in the supplementation group, while levels increased in the control group [39]. As previously suggested, although dairy consumption may not reduce circulating TNF-α concentrations, it could increase soluble TNF-α receptors, which bind TNF-α and help attenuate its activity [38]. However, our study cannot confirm the role of TNF-α receptors in response to increased dairy intake.

### 4.4. Strengths and Limitations

Strengths of this pilot intervention trial include the use of a real-world community exercise program, and dietary verification using ASA24 recall assessments. The study was a pilot intervention and, thus, limited by its short duration (8 weeks) and absence of exclusion criteria regarding medication use, which warrants caution when interpreting the findings. While excluding certain medications could offer methodological advantages, implementing such a design would have been challenging and might have reduced the study’s relevance and applicability to the target population of older adults. In addition, since the study was intended as a pilot, sample size was not calculated a priori. After using G*Power (version 3.1) to calculate power a posteriori for repeated measures analysis of variance (repeated measures, within-between interaction), we found that for an effect size 0.20 and a significance level of 0.05, the calculated power for 48 participants, 3 groups and 2 measurements/times was 65%. Thus, our modest sample size may have played a role in limiting the power to detect interaction effects. Other limitations and potential sources of bias in this pilot study include the absence of a dietary control to GY, seasonal effects, and lack of participant blinding.

## 5. Conclusions

This 8-week pilot intervention with GY supplementation effectively increased dietary protein and calcium intake, resulting in modest changes in bone turnover markers among older adult exercisers. These changes included an increase in CTX-I and a stabilization of DKK-1 in the exercisers consuming GY, which was opposite to what was observed in the non-exercisers. However, the implications of these limited findings for long-term skeletal health remain unclear due to the lack of differential treatment effects in bone formation markers between groups. Significant time x group interactions were detected in pro-inflammatory cytokines, reflecting non-significant decreases in IL-1β and IL-6 among the exercisers, whereas in non-exercisers, GY supplementation was associated with an increase in IL-1β and a reduction in IL-6—an interesting observation that requires further investigation. Importantly, the modest results of this short intervention should be interpreted cautiously due to pilot design and statistical limitations. Future studies should consider increasing the sample size and using more sensitive biomarkers to detect subtle group differences. Finally, longer interventions are warranted to determine whether whole-food dairy proteins can meaningfully influence skeletal and immune health in this population.

## Figures and Tables

**Table 1:** Daily dietary intake at weeks 0 and 8 for participants in the Greek yogurt exercisers (*n* = 18, 12 females), Greek yogurt non-exercisers (*n* = 15, 13 females), and no yogurt exercisers (*n* = 15, 10 females).

		Week 0	Week 8	Time	Group	Time × Group
Energy Intake (kcal)	GYEX	1883 ± 400	1926 ± 505 *	0.333	0.093	0.041
	GYNE	1502 ± 403	1783 ± 381 *			
	NYEX	1832 ± 327	1695 ± 345 *			
Carbohydrate Intake (g/kg)	GYEX #	3.35 ± 0.73	3.46 ± 0.81	0.101	0.036	0.057
	GYNE #	2.57 ± 0.67	3.07 ± 0.58			
	NYEX	3.06 ± 0.71	2.95 ± 0.72			
Protein Intake (g/kg)	GYEX ##	1.22 ± 0.35	1.30 ± 0.30 *	0.019	0.003	0.015
	GYNE	0.87 ± 0.25	1.10 ± 0.23 *			
	NYEX	1.02 ± 0.15	0.97 ± 0.27			
Fat intake (g)	GYEX	67.9 ± 18.9	65.6 ± 22.4	0.884	0.55	0.195
	GYNE	51.6 ± 18.2	59.2 ± 16.5			
	NYEX	69.9 ± 16.4	63.2 ± 15.1			
Vitamin D Intake (mcg)	GYEX	8.66 ± 5.0	11.38 ± 5.15 *	0.604	0.445	0.052
	GYNE	8.48 ± 6.09	10.00 ± 4.63 *			
	NYEX	9.64 ± 5.15	6.86 ± 4.49			
Calcium Intake (mg)	GYEX ##	1010 ± 405	1522 ± 1156 *	<0.001	0.003	<0.001
	GYNE	734 ± 296	1156 ± 276 *			
	NYEX	1137 ± 320	932 ± 339			

Values are means ± standard deviations. GYEX = Greek yogurt exercisers group; GYNE = Greek yogurt non-exercisers group. NYEX = no yogurt exercisers group. Significant effects (*p* ≤ 0.05) are bolded. * denotes significant within-group post hoc difference (*p* ≤ 0.05) between weeks 0 and 8 following significant interaction. # denotes significant between-group post hoc difference (*p* ≤ 0.05) between GYEX and GYNE following significant main group effect. ## denotes significant between-group post hoc difference (*p* ≤ 0.05) between GYEX and both GYNE and NYEX following significant main group effect.

**Table 2:** Changes in body mass, body mass index (BMI), and body fat at weeks 0 and 8 for participants in the Greek yogurt exercisers (*n* = 18, 12 females), Greek yogurt non-exercisers (*n* = 15, 13 females), and no yogurt exercisers (*n* = 15, 10 females).

		Week 0	Week 8	Time	Group	Time × Group
Body Mass (kg)	GYEX	72.4 ± 15.6	73.0 ± 15.8	0.033	0.222	0.421
	GYNE	76.5 ± 15.5	77.4 ± 15.9			
	NYEX	75.3 ± 14.8	75.8 ± 14.9			
BMI (kg/m^2^)	GYEX	26.5 ± 5.1	26.7 ± 5.1	0.135	0.132	0.370
	GYNE	29.6 ± 5.9	29.9 ± 5.9			
	NYEX	27.6 ± 5.2	27.8 ± 5.3			
Body Fat (%)	GYEX	31.7 ± 8.6	31.9 ± 8.7	0.599	0.006	0.542
	GYNE ##	39.3 ± 7.2	39.0 ± 7.4			
	NYEX	34.7 ± 8.4	34.4 ± 8.1			

Values are means ± standard deviations. GYEX = Greek yogurt exercisers group; GYNE = Greek yogurt non-exercisers group. NYEX = no yogurt exercisers group. Significant effects (*p* ≤ 0.05) are bolded. ## denotes significant between-group post hoc difference (*p* ≤ 0.05) between GYNE and both GYEX and NYEX following significant main group effect.

**Table 3:** Circulating concentrations of markers of bone turnover at weeks 0 and 8 for participants in the Greek yogurt exercisers (*n* = 18, 12 females), Greek yogurt non-exercisers (*n* = 15, 13 females), and no yogurt exercisers (*n* = 15, 10 females).

		Week 0	Week 8	Time	Group	Time × Group Interaction
OC (pg/mL)	GYEX	3074 ± 1245	2839 ± 955	0.111	0.733	0.709
	GYNE	2261 ± 1055	2193 ± 1248			
	NYEX	3430 ± 1900	3199 ± 1742			
P1NP (ng/mL)	GYEX	1075 ± 382	1064 ± 411	0.102	0.680	0.482
	GYNE	949 ± 460	873 ± 504			
	NYEX	1075 ± 382	1064 ± 411			
CTX-I (pg/mL)	GYEX	0.26 ± 0.16	0.29 ± 0.17 *	0.426	0.500	0.038
	GYNE	0.18 ± 0.18	0.16 ± 0.11			
	NYEX	0.21 ± 0.19	0.19 ± 0.15			
PTH (pg/mL)	GYEX	17.3 ± 9.2	16.9 ± 7.3	0.080	0.854	0.738
	GYNE	18.0 ± 14.3	18.2 ± 14.7			
	NYEX	19.0 ± 10.9	17.4 ± 9.7			
OPG (pg/mL)	GYEX	493 ± 233	465 ± 190	0.002	0.349	0.169
	GYNE	563 ± 197	520 ± 231			
	NYEX	411 ± 101	411 ± 116			
RANKL (pg/mL)	GYEX	27.0 ± 23.5	28.0 ± 22.6	0.837	0.653	0.591
	GYNE	23.8 ± 16.0	26.0 ± 16.4			
	NYEX	39.3 ± 25.1	32.7 ± 19.6			
SOST (pg/mL)	GYEX	581 ± 229	576 ± 206	0.360	0.895	0.675
	GYNE	565 ± 187	555 ± 204			
	NYEX	506 ± 141	475 ± 136			
DKK-1 (pg/mL)	GYEX	903 ± 180	880 ± 195	0.018	0.254	0.030
	GYNE	749 ± 215	850 ± 226 *			
	NYEX	670 ± 221	733 ± 155			

Values are means ± standard deviations. GYEX = Greek yogurt exercisers group; GYNE = Greek yogurt non-exercisers group. NYEX = no yogurt exercisers group. OC = Osteocalcin; P1NP = Procollagen Type I *N*-terminal Propeptide; CTX-I = *C*-Terminal Telopeptide of Type I Collagen; PTH = Parathyroid Hormone; OPG = Osteoprotegerin; RANKL = Receptor Activator of Nuclear Factor κB Ligand; SOST = Sclerostin; DKK-1 = Dickkopf-1. Significant effects (*p* ≤ 0.05) are bolded. * denotes significant within-group post hoc difference (*p* ≤ 0.05) between weeks 0 and 8 following significant interaction.

**Table 4:** Circulating concentrations of pro-inflammatory cytokines, including interleukin 1-beta (IL-1β) interleukin 6 (IL-6) and tumor necrosis factor alpha (TNF-α), at weeks 0 and 8 for Greek yogurt exercisers (GYEX, *n* = 18, 12 females), Greek yogurt non-exercisers (GYNE, *n* = 15, 13 females), and no yogurt exercisers (NYEX, *n* = 14, 10 females).

		Week 0	Week 8	Time	Group	Time × Group Interaction
IL-1β (pg/mL)	GYEX	0.33 ± 0.18	0.20 ± 0.18	0.107	0.378	0.043
	GYNE	0.24 ± 0.10	1.13 ± 2.15 *			
	NYEX	0.26 ± 0.09	0.46 ± 0.81			
IL-6 (pg/mL)	GYEX	0.54 ± 0.81	0.29 ± 0.16	<0.001	0.076	0.023
	GYNE	1.56 ± 2.55	0.21 ± 0.10 *			
	NYEX	1.10 ± 1.88	0.23 ± 0.09			
TNF–α (pg/mL)	GYEX	2.84 ± 1.06	2.68 ± 1.10	0.408	0.971	0.905
	GYNE	2.43 ± 0.90	2.43 ± 1.01			
	NYEX	2.69 ± 1.00	2.68 ± 1.04			

Values are means ± standard deviations. GYEX = Greek yogurt exercisers group; GYNE = Greek yogurt non-exercisers group. NYEX = no yogurt exercisers group. IL-1β = Interleukin 1-beta; IL-6 = interleukin; TNF-α = Tumor Necrosis Factor-alpha. Significant effects (*p* ≤ 0.05) are bolded. * denotes significant within-group post hoc difference (*p* ≤ 0.05) between weeks 0 and 8 following significant interaction.

## Data Availability

The data supporting the findings of this study are available from the corresponding author PK upon reasonable request.

## Abbreviations

The following abbreviations are used in this manuscript:

GY	Greek Yogurt
GYEX	Greek Yogurt + Exercise group
NYEX	No Yogurt Exercise group
GYNE	Greek Yogurt No Exercise group
BMD	Bone Mineral Density
IL-1β	Interleukin-1 beta
IL-6	Interleukin-6
TNF-α	Tumor Necrosis Factor-alpha
REB	Research Ethics Board
TCPS2	Tri-Council Policy Statement: Ethical Conduct for Research Involving Humans (2nd edition)
ASA24	Automated Self-Administered 24-Hour Dietary Recall
BIA	Bioelectrical Impedance Analysis
LBM	Lean Body Mass
FM	Fat Mass
%BF	Percent Body Fat
P1NP	Procollagen Type I *N*-terminal Propeptide
CTX-I	*C*-Terminal Telopeptide of Type I Collagen
OC	Osteocalcin
OPG	Osteoprotegerin
RANKL	Receptor Activator of Nuclear Factor κB Ligand
SOST	Sclerostin
DKK-1	Dickkopf-1
PTH	Parathyroid Hormone
ELISA	Enzyme-Linked Immunosorbent Assay
ANOVA	Analysis of Variance
FDR	False Discovery Rate
SEM	Standard Error of the Mean
%CV	Coefficient of Variation (percentage)
